# Syringaresinol Attenuates α-Melanocyte-Stimulating Hormone-Induced Reactive Oxygen Species Generation and Melanogenesis

**DOI:** 10.3390/antiox13070876

**Published:** 2024-07-21

**Authors:** Kyuri Kim, Jihyun Yoon, Kyung-Min Lim

**Affiliations:** College of Pharmacy, Ewha Womans University, Seoul 03760, Republic of Korea; kyuri0912@ewha.ac.kr (K.K.); wlgus3398@ewhain.net (J.Y.)

**Keywords:** (+)-syringaresinol, ginseng berry, antioxidant, melanogenesis, 3D skin model

## Abstract

Ginseng has been utilized for centuries in both the medicinal and cosmetic realms. Recent studies have actively investigated the biological activity of ginseng berry and its constituents. (+)-Syringaresinol [(+)-SYR], an active component of ginseng berry, has been demonstrated to have beneficial effects on the skin, but its potential impact on skin pigmentation has not been fully explored. Here, the antioxidant and anti-pigmentary activity of (+)-SYR were evaluated in B16F10 murine melanoma cells and in an artificial human pigmented skin model, Melanoderm™. A real-time PCR, Western blotting, immunofluorescence staining, and histochemistry staining were conducted to confirm the effects of (+)-SYR on pigmentation. (+)-SYR reduced melanogenesis and dendrite elongation in α-melanocyte-stimulating hormone (α-MSH)-primed B16F10 cells with low cytotoxicity. (+)-SYR suppressed the expression of melanogenic genes, namely tyrosinase (TYR), tyrosinase-related protein 1 (TRP-1), and tyrosinase-related protein 2 (TRP-2). Notably, (+)-SYR attenuated α-MSH-induced cytosolic and mitochondrial reactive oxygen species (ROS) generation, which was attributable at least in part to the suppression of NADPH oxidase-4 (NOX 4) expression. Finally, the brightening activities of (+)-SYR were verified using Melanoderm™, underscoring the potential of ginseng berry and (+)-SYR as functional ingredients in skin-brightening cosmetics.

## 1. Introduction

Panax ginseng has a long history of therapeutic use and is widely recognized for its utility in reducing fatigue and improving general health [1]. Extensive research has identified various active components in ginseng, including organic acids, flavonoids, saponins, peptides, and minerals [2]. Furthermore, not only the root but also the fruit of *P. ginseng*, namely ginseng berry, has pharmacological properties, such as antioxidant and anti-cancer activities [3,4]. Notably, one of the active components of ginseng berry, (+)-syringaresinol ((+)-SYR), shows potent anti-inflammatory and anti-aging activities, drawing attention for its utility as a cosmetic ingredient [5]. (+)-SYR is a lignan, a polyphenol, an aromatic ether, and a furofuran. (+)-SYR is not only found in ginseng but also present in various plants. Previous research indicates that (+)-SYR occurs in the roots of *Rubia philippinensis* and the dried stems of *Acanthopanax divaricatus,* which exhibits anti-inflammatory effects and vasorelaxant effects through its interaction with NO synthase [6,7]. Recently, Kim et al. showed that (+)-SYR from Korean ginseng berry exerts anti-pigmentary effects through FoxO3a regulation [8], but its anti-melanogenic effects with respect to melanogenesis and antioxidant activity have not yet been fully elucidated.

Melanin production occurs inside melanocyte melanosomes and has a significant influence over the pigmentation of human skin, hair, and eyes [9]. However, excessive melanin production can result in various cosmetic concerns, such as freckles, solar lentigo, melasma, ephelides, and other forms of skin pigmentation abnormalities [10]. Melanogenesis occurs within melanocytes situated in the basal layer of the epidermis. These melanocytes extend dendrites to transport melanin pigments to adjacent keratinocytes [11,12]. This process is catalyzed by various types of melanogenesis-related enzymes: tyrosinase, tyrosinase-related protein 1 (TRP-1), and tyrosinase-related protein 2 (TRP-2) [13]. Melanin synthesis may be triggered by extrinsic and intrinsic factors, including ultraviolet (UV) radiation or α-melanocyte-stimulating hormone (α-MSH) [14,15]. The overabundance of reactive oxygen species (ROS) significantly contributes to the excessive production of melanin, which serves to safeguard DNA and other essential endogenous molecules from ROS [16].

As the demand for skin-brightening products rapidly grows, there is an increasing demand for environmentally friendly cosmetic ingredients originated from natural substances [17]. Various materials, such as kojic acid, hydroquinone, and arbutin, have been introduced for their anti-pigmentation properties [18]. Kojic acid inhibits tyrosinase activity in melanin synthesis and hinders the final stage of melanin production [19]. Also, hydroquinone (HQ), a prescription medication for skin care, and arbutin, a D-glucopyranoside of hydroquinone, inhibit tyrosinase activity. Despite their wide uses in skin brightening, these substances can cause side effects such as skin irritation, contact dermatitis, and hyperpigmentation [20,21]. Against this background, the discovery of skin-brightening agents free from adverse effects is high in demand.

Recently, antioxidants have been gaining attention as safe and effective skin brightening agents. Antioxidants such as Vitamin C [22], resveratrol [23], and ferulic acid [24] have demonstrated effectiveness in skin brightening. Antioxidants manifest skin-brightening effects by either reducing the activity of tyrosinase or preventing the oxidative polymerization of melanin intermediates [25]. Recent efforts have focused on the discovery of natural antioxidants with improved activity and safety.

In this study, we examined the antioxidant and anti-pigmentation activities of (+)-SYR using B16F10F10 cells and an artificial human pigmented skin model, Melanoderm™. We further investigated the mechanism responsible for the anti-melanogenic and antioxidant activities of (+)-SYR to explore the potential of ginseng berry and (+)-SYR for use in skin-brightening cosmetics.

## 2. Materials and Methods

### 2.1. Materials

(+)-SYR (purity > 98%) was purchased from Q-mine (Seoul, Korea). A stock solution (10 mg/mL) was prepared with DMSO purchased from Sigma-Aldrich (St Louis, MO, USA).

### 2.2. Cell Culture 

The B16F10 cell line was purchased from ATCC (Manassas, VA, USA), and the cells were maintained in the culture media. At 80% cell confluence, adherent cells were detached with a solution of 0.05% trypsin (Hyclone, South Logan, UT, USA).

### 2.3. Melanin Assay and Cell Viability Assay

To quantify the melanin content, B16F10F cells were cultured in 48-well plates. Cells were treated with indicated concentrations of (+)-SYR in the culture media containing 0.2 μM of α-Melanocyte Stimulating Hormone (α-MSH) for 48 h. Cells without α-MSH treatment were used as negative controls, while cells treated with 50 μg/mL arbutin were used as positive controls. The cells were lysed in a solution of 1 N NaOH at 60 °C for 1 h in the dark. The melanin content was quantified at a wavelength of 405 nm using a microplate reader (Spectra max 190, Molecular Devices, Sunnyvale, CA, USA), as in a previous study [26]. Cell viability was assessed using 3-(4,5-dimethylthiazol-2-yl)-2,5-diphenyltetrazolium bromide (MTT). B16F10 cells were incubated with 0.5 mg/mL MTT solution in DMEM for 2 h at 37 °C. The supernatant was measured at 540 nm. All measurements were performed in triplicate.

### 2.4. Mushroom Tyrosinase Inhibition Assay

A mushroom tyrosinase assay was conducted to evaluate the inhibitory effect of (+)-SYR on tyrosinase activity. For the assay, 0.03% l-tyrosine in 0.1 M potassium phosphate was added to 250 units of mushroom tyrosinase, and 0.2% l-DOPA in 0.1 M potassium phosphate was mixed with 50 units of mushroom tyrosinase. In other words, different units of mushroom tyrosinase were added to each l-tyrosine and l-DOPA solution. The com-pound dissolved in DMSO was added and incubated at 37 °C for the specified duration. Absorbance was measured at 475 nm using a microplate reader (Spectra max 190, Molec-ular Devices, Sunnyvale, CA, USA).

### 2.5. DPPH Assay and Intracellular ROS Level

A 100 μM stock solution of DPPH dissolved in ethanol was dispensed at 247.5 μL into each well of a 96-well plate, with the sample added to achieve a final reaction volume of 250 μL according to a previously described method [27]. Ascorbic acid at a concentration of 30 μM served as the positive control. The reaction mixture was then incubated at room temperature for 0, 15, 30, 60, and 180 min in the absence of light. Absorbance readings were taken at 517 nm using a microplate reader. Data analysis was conducted using the following equation:

$$\mathrm{Radical}\;\mathrm{Savenging}\;\%=100-\left\{\frac{\left(Abs\;sample-Abs\;blank\right)*100}{Abs\;Control}\right\}$$

ROS production was assessed using a fluorescence assay enhanced by 2′,7′-dichlorofluoresceindiacetate (DCF-DA, Eugene, OR, USA). B16F10 cells treated with (+)-SYR for 24 h were stained with DCF-DA for 10 min. Ascorbic acid at a concentration of 30 µM served as the positive control. Subsequently, images of the treated cells were captured using the Softmax 5.2 program and an Axiovert 200M microscope (Carl Zeiss, Land Baden-Württemberg, Germany).

### 2.6. Mitochondrial Superoxide Assay 

To evaluate mitochondrial superoxide levels in B16F10 cells, the cells were grown in 4-well chambers until they reached 80% confluence. They were then treated with α-MSH and (+)-SYR for 24 h. MitoSOX (Invitrogen, Waltham, MA, USA) and Mitotracker green (Invitrogen, MA, USA) were applied to cover the cells on the coverslip as per the manufacturer’s instructions. The cells were allowed to incubate with MitoSOX for 30 min at 37 °C and 5% CO_2_. Following the incubation, the cells were gently washed twice with Hank’s Buffered Salt Solution (HBSS) buffer and mounted with fluoromount-G. Confocal microscopy analysis was conducted within 2 h of staining.

### 2.7. RNA Sample Preparation and Real-Time PCR

After 24 h of exposure to (+)-SYR, B16F10 cells were rinsed twice with phosphate-buffered saline and lysed using Trizol (Invitrogen, MA, USA). Following the addition of chloroform, samples were centrifuged at 12,000 rpm for 10 min. The aqueous phase was then combined with isopropanol, and RNA pellets were precipitated by centrifugation (12,000 rpm, 15 min, 4 °C). Subsequently, RNA pellets were washed with 70% ethanol and dissolved in RNase-free, diethylpyrocarbonate (DEPC)-treated water (Waltham, MA, USA). The RNA concentration was determined by measuring the optical density at 260 nm using a spectrophotometer (NanoDrop Technologies, Inc., Wilmington, DE, USA). A quantitative real-time PCR was employed to assess the relative expression levels of mRNA. cDNA synthesis was conducted using 1250 ng of total RNA with oligo(dT) (Bioelpis, Seoul, Republic of Korea). Each reaction utilized a SYBR Green PCR master mix and a StepOnePlusTM Real-time PCR machine (Applied Biosystems, Warrington, UK). The β-actin gene was used as a house-keeping gene to normalize the mRNA levels of the target genes. The sequence of primers was as follows:
Forward β-actin5′-AGG GAA ATC GTG CGT GAC AT-3′Reverse β-actin5′-GGA AAA GAG CCT CAG GGC AT-3′Forward Tyrosinase5′-GGG CCC AAA TTG TAC AGA GA-3′Reverse Tyrosinase5′-ATG GGT GTT GAC CCA TTG TT-3′Forward TRP-15′-GTT CAA TGG CCA GGT CAG GA-3′Reverse TRP-15′-CAG ACA AGA AGC AAC CCC GA-3′Forward TRP-25′-TTA TAT CCT TCG AAA CCA GGA-3′R0everse TRP-25′-GGG AAT GGA TAT TCC GTC TTA-3′

### 2.8. Western Blot Analysis

B16F10 cells were cultured in 6-well plates for 24 h and treated with varying concentrations of (+)-SYR and 0.2 µM α-MSH for 24 and 48 h. Post treatment, cells were homogenized in lysis buffer containing RIPA buffer, 1 mM phenyl methane sulfonyl fluoride (PMSF), and 1% protease inhibitor cocktail (PIC) at 4 °C for 20 min, then scraped and centrifuged at 12,000 rpm for 10 min at 4 °C. The supernatant was collected and the protein concentration was determined using a BCA assay. Proteins were denatured by boiling at 95 °C for 5 min, separated by 10% sodium dodecyl sulfate polyacrylamide gel electrophoresis (SDS-PAGE), and transferred to nitrocellulose membranes (Amersham, Buckinghamshire, UK). Membranes were blocked with 5% BSA at room temperature for 2 h, then incubated with primary antibodies against target proteins in 5% BSA at 4 °C for 18 h. After washing, membranes were incubated with HRP-conjugated secondary antibodies (KPL, Gaithersburg, MD, USA) for 1 h at room temperature. Bound antibodies were detected using ECL Western blotting reagents (Amersham Biosciences, Little Chalfont, UK) and visualized with Amersham Imager 600 (GE Healthcare Life Sciences, UK). TYR antibody (Abcam, Cambridge, UK), TRP-1 anti-body (Abcam, Cambridge, UK), TRP-2 antibody (Abcam, Cambridge, UK), NOX4 antibody (Invitrogen, MA, USA), β-actin antibody (Santa Cruz Biotechnology, Santa Cruz, CA, USA), and GAPDH (Abcam, Cambridge, UK) were utilized as controls.

### 2.9. Brightening Assay with a Pigmented Human Epidermal 3D Skin Model, Melanoderm™

Melanoderm™ (MatTek, MA, USA) is a three-dimensional human epidermis composed of normal human epidermal keratinocytes and normal human melanocytes. Following a 24 h pre-incubation period, (+)-SYR was administered to the tissues every other day for a duration of 14 days. On the 14th day of the experiment, the tissues were fixed using phosphate-buffered formalin and subsequently stained with hematoxylin and eosin (H&E) as well as Fontana–Masson (FM). Quantitative analysis of the stained tissue was conducted using Image J software (https://imagej.net/ij/, accessed on 1 September 2023).

### 2.10. Statistical Analysis

The results are presented as the mean ± standard error of the mean (SEM) from three or more independent experiments. Statistical analyses were conducted using Student’s *t*-test. Data were considered statistically significant if the *p*-value was less than 0.05.

## 3. Results

### 3.1. The Effect of (+)-SYR on Melanogenesis and Cell Viability of B16F10 Cells

**Figure 1:**
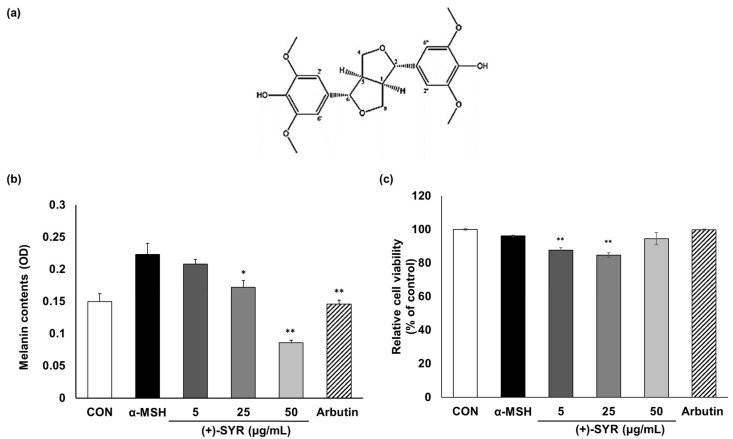
The effect of (+)-SYR on the cell viability and melanin contents of α-MSH (0.2 µM)-treated B16F10 cells. (**a**) The structure of (+)-SYR. (**b**) Melanin contents were measured by melanin assay. (**c**) Cell viability was determined by MTT assay. Data are presented as the mean ± SD (n = 4, ** *p* < 0.01 and * *p* < 0.05 compared with the α-MSH-treated group).

With α-MSH stimulation, a range of concentrations of (+)-SYR (Figure 1a), from 1 to 50 µg/mL, or the positive control, beta-arbutin (50 µg/mL), were treated with a murine melanoma cell line, B16F10 cells, for 48 h. (+)-SYR inhibited the melanin content in the cells in a dose-dependent manner (Figure 1b), without significant cytotoxicity, as cell viability remained above 80% (Figure 1c). 

### 3.2. Effect of (+)-SYR on Cell Morphology of B16F10

**Figure 2:**
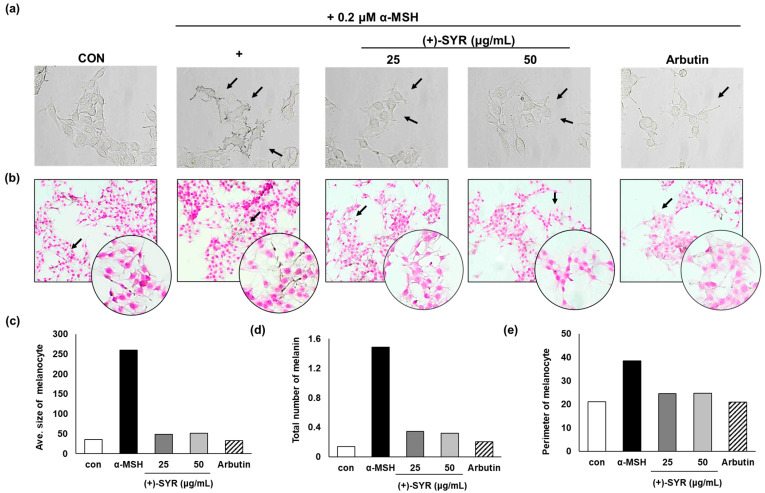
Effects of (+)-SYR on the dendrite formation in B16F10 cells. (**a**) Morphological changes in B16F10 were observed after (+)-SYR treatment for 24 h. (**b**) Morphological changes were observed under optical microscopy (100×) after Fontana–Masson staining (black arrows indicate the stained melanocyte with extended dendrites). (**c**) Average size of melanocyte, (**d**) total number of melanin, and (**e**) perimeter of melanocytes were analyzed by Image J software (https://imagej.net/ij/, accessed on 1 September 2023), n = 3.

The anti-melanogenic activities of (+)-SYR were further confirmed through microscopic observation. The visual analysis revealed that (+)-SYR significantly reduced the dendrite formation stimulated by α-MSH (Figure 2a). Additionally, microscopic examination using Fontana–Masson (FM) staining, which stains melanin dark black, further supported that (+)-SYR attenuated the dendrite formation in B16F10F10 cells (Figure 2b). The data were analyzed using Image J software (https://imagej.net/ij/, accessed on 1 September 2023), and the results indicated that the average size of melanocytes (Figure 2c), the total melanin content (Figure 2d), and the perimeter of melanocytes (Figure 2e) were all reduced consistently with the microscopic observations, demonstrating that (+)-SYR can suppress α-MSH-stimulated melanocyte activation.

### 3.3. (+)-SYR Downregulates the Expression of Melanogenic Genes without Affecting Enzymatic Activity

**Figure 3:**
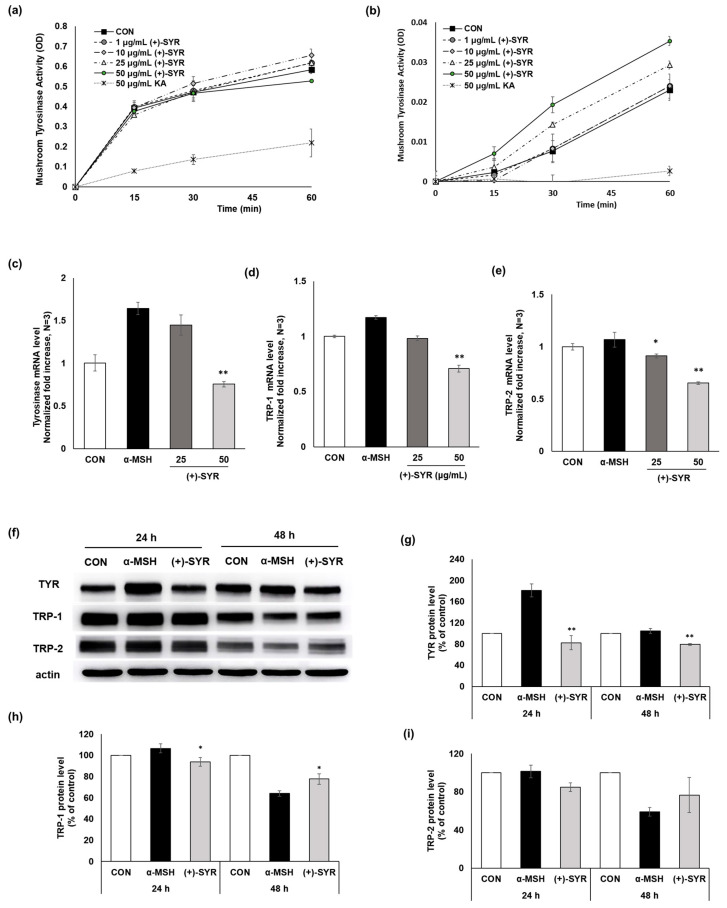
The effect of (+)-SYR on the expression of melanogenic-related genes. Tyrosinase enzymatic activity was measured using mushroom tyrosinase assay with (**a**) l-tyrosine and (**b**) l-DOPA as substrates. mRNA expression levels of (**c**) tyrosinase; (**d**) TRP-1; (**e**) and TRP-2 in B16F10 cells were determined by real-time PCR. The cells were treated with SYR (25, 50 µg/mL) for 24 h. (**f**) Tyrosinase, TRP-1, and TRP-2 protein levels were determined by Western blotting. The representative is the first result (#4) (out of n = 4) from Supplementary Materials. (**g**–**i**) quantitation of TYR, TRP-1, and TRP-2 was analyzed by Image J software (https://imagej.net/ij/, accessed on 1 September 2023). The cells were treated with (+)-SYR at a concentration of 50 µg/mL with 0.2 µM of α-MSH for the indicated times. Data are presented as the mean ± SD (n = 3 or 4, ** *p* < 0.01 and * *p* < 0.05 compared with the α-MSH-treated group).

Tyrosinase (TYR) serves as a pivotal and rate-limiting enzyme in melanogenesis. An assay using mushroom tyrosinase indicated that the enzymatic activity of tyrosinase itself remained unaffected by (+)-SYR (Figure 3a,b). qPCR was conducted to assess alterations in the expression levels of melanogenic genes, including TYR, tyrosinase-related protein 1 (TRP-1), and tyrosinase-related protein 2 (TRP-2). TYR expression was upregulated following α-MSH treatment alone, but a 24 h co-treatment of (+)-SYR and α-MSH reduced TYR expression (Figure 3c). The expressions of TRP-1 and TRP-2 were also reduced by (+)-SYR treatment (Figure 3d,e). Furthermore, we confirmed the protein changes in melanin synthesis proteins through the Western blot assay. As a result, the protein expression of TYR was prominently increased when the cells were treated α-MSH alone. The co-treatment of (+)-SYR and α-MSH significantly reduced the protein expression of TYR after a 24 h application (Figure 3f,g). On the other hand, exposure to (+)-SYR for 48 h did not significantly change the protein expression of TYR. Similarly, the inhibition of melanin synthesis enzyme protein levels by (+)-SYR was also confirmed in TRP-1 and TRP-2 expression (Figure 3f,h,i). These findings further support that the anti-pigmentary effect of (+)-SYR involves the inhibition of the expression of melanin synthesis enzymes.

### 3.4. (+)-SYR Suppresses ROS Production in α-MSH-Stimulated B16F10

**Figure 4:**
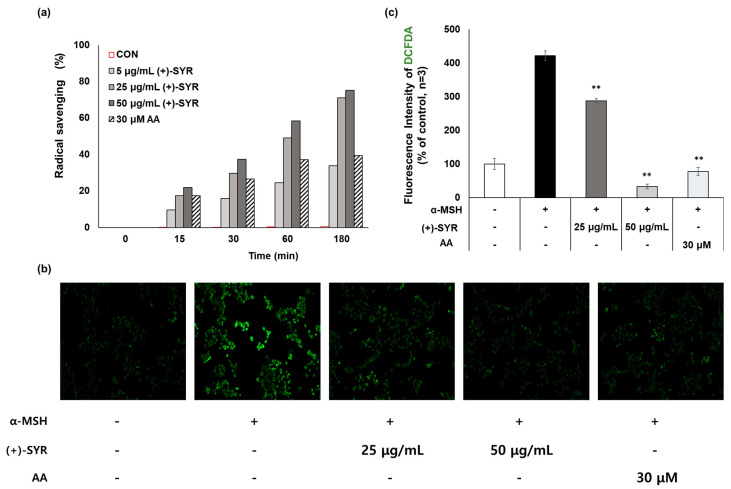
Antioxidant activity of (+)−SYR was measured by (**a**) DPPH radical scavenging activity assay and (**b**) DCF-DA fluorescence staining (200×); (**c**) fluorescence intensity of DCF-DA was obtained by Image J software (https://imagej.net/ij/, accessed on 1 September 2023), n = 3. α-MSH (0.2 µM)-stimulated B16F10 cells were treated with (+)-SYR for 24 h. An amount of 30 µM of ascorbic acid (AA) was used as the positive control for this experiment. Data are presented as the mean ± SD (n = 3, ** *p* < 0.01 compared with the α-MSH-treated group.

Overexposure to UV radiation or free radicals frequently leads to skin damage, including skin aging and pigmentation. According to previous studies, free radicals, along with prolonged UVA and UVB exposure, significantly increase levels of hormones like β-lipotropin (β-LPH) and α-MSH. These hormones induce an increase in melanin production in human melanocytes via the activation of cAMP-dependent protein kinase A (PKA) and MITF. Upon MITF activation, phosphorylated MITF facilitates the synthesis of tyrosinase (TYR) and associated proteins, stimulating melanin production and synthesis [28]. Therefore, many antioxidants have the potential to suppress melanogenesis [29,30,31]. Here, we evaluated the antioxidant activity of (+)-SYR by DPPH (1,1-diphenyl-2-pricrylhydrazyl) assay and DCF-DA (2′,7′-dichlorofluoresceindiacetate) assay. Ascorbic acid (AA), a well-known antioxidant, was used as positive control for this study. In the DPPH assay, which is simple and one of the most widely used methods for determining antioxidant activity based on spectrophotometric measurement [32], (+)-SYR showed potent antioxidant activity (Figure 4a). We further confirmed the antioxidant activity of (+)-SYR in B16F10 cells with DCF-DA (Figure 4b). The fluorescence intensity of DCF-DA was analyzed using Image J software (https://imagej.net/ij/, accessed on 1 September 2023) (Figure 4c) and the results showed that (+)-SYR exhibited remarkable scavenging effects against α-MSH-stimulated ROS compared to the positive control, ascorbic acid. These findings suggest that the antioxidant activity of (+)-SYR could contribute, at least in part, to its anti-melanogenesis effects.

**Figure 5:**
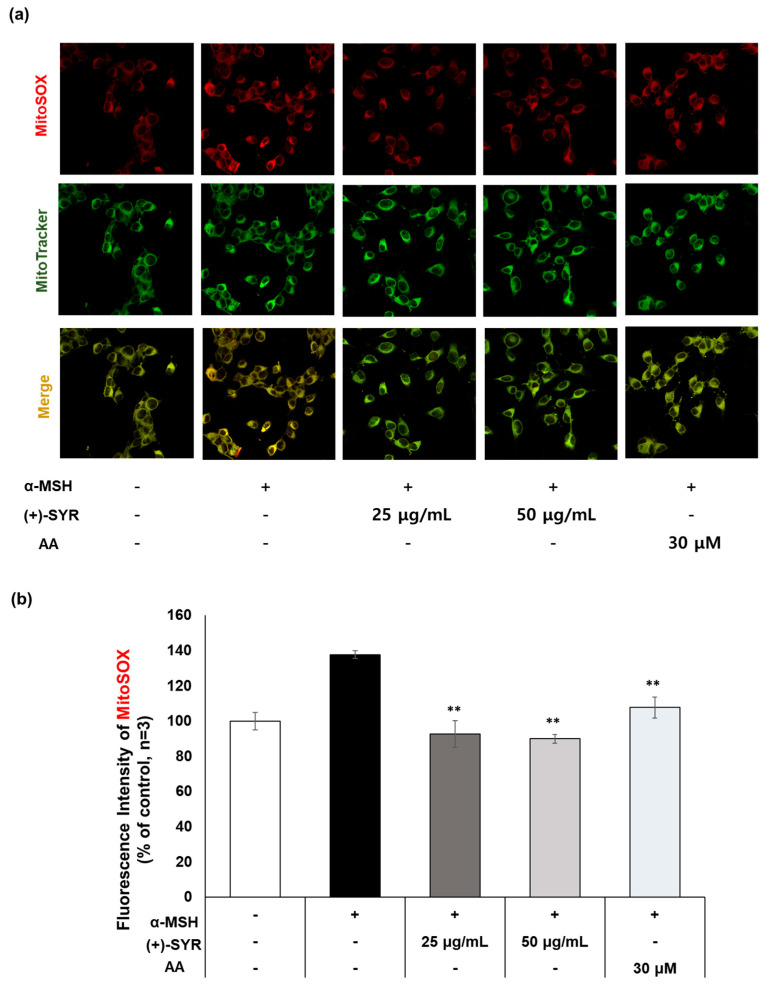
Antioxidant effect of (+)−SYR using fluorescent probes. (**a**) Cells stained with MitoSOX red and MitoTracker Green. Representative images were obtained by confocal microscopy (400×). (**b**) Fluorescence intensity of MitoSOX red was obtained by image J software (https://imagej.net/ij/, accessed on 1 September 2023). (n = 3). α-MSH (0.2 µM)-stimulated B16F10 cells were treated with SYR for 24 h. An amount of 30 µM of ascorbic acid (AA) was used as the positive control for this experiment. Data are presented as the mean ± SD (n = 3, ** *p* < 0.01 compared with the α-MSH-treated group).

To check if (+)-SYR could attenuate ROS generation in mitochondria, the fluorescent probe MitoSOX Red, which is used to image the superoxide in live cell mitochondria, was employed (Figure 5a). As a result, in the group treated with α-MSH alone, the fluorescence intensity of MitoSOX was significantly increased, while (+)-SYR suppressed the signal significantly. Especially in the merged images, the antioxidant effect of (+)-SYR was clearly evident. These results demonstrated the ROS-reducing effect of (+)-SYR in cytosol and mitochondria. Interestingly, the fluorescence intensity of DCF-DA was more noticeably reduced by (+)-SYR than that of MitoSOX Red, suggesting that the antioxidant effect of (+)-SYR acts more on cytosolic ROS than on mitochondrial ROS.

### 3.5. (+)-SYR Suppresses NOX4 in α-MSH Stimulated B16F10

**Figure 6:**
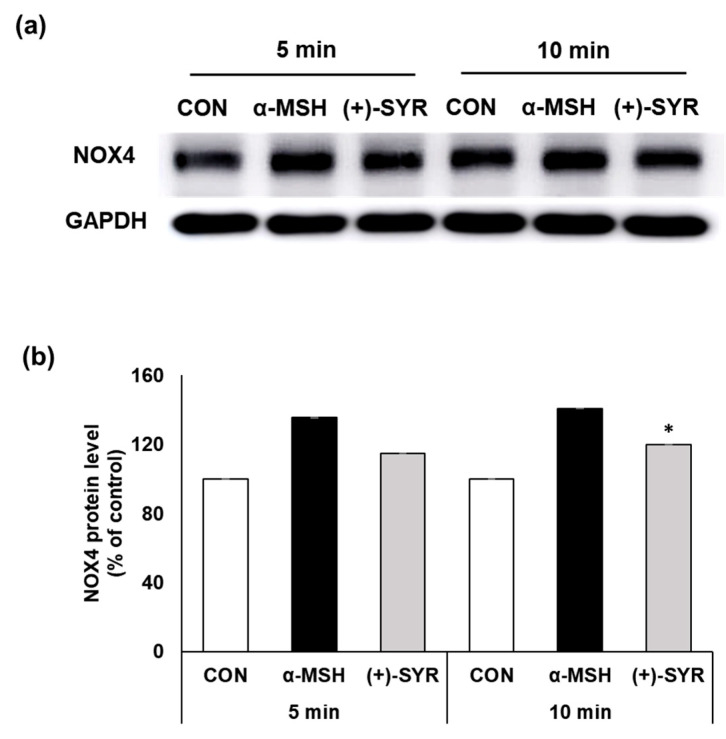
(+)-SYR suppresses ROS production in α-MSH stimulated B16F10. (+)-SYR suppresses ROS production in α-MSH stimulated B16F10. (**a**) NOX4 protein levels were determined by Western blot assay. The representative is the first result (n1) (out of n = 4) from Supplementary Materials. The cells were treated with (+)-SYR at a concentration of 50 µg/mL with 0.2 µM of α-MSH for the indicated times. (**b**) Quantitation of NOX4 protein level. The quantitation of the Western blot band was analyzed by Image J software (https://imagej.net/ij/, accessed on 1 September 2023). Data are presented as the mean ± SE (n = 4, * *p* < 0.05 compared with the α-MSH-treated group).

Among the pathways of ROS generation in melanocytes, NADPH oxidases (NOXs) play a key role in cytosolic ROS generation upon exposure to α-MSH [26]. According to previous studies that examined the levels of NOX expression in normal human melanocytes (NHMs) and melanoma cell lines, NOX4 was found to play a major role in the melanocyte lineage [33]. Supporting this, in B16F10 cells, α-MSH induced a significant increase in NOX4 gene and protein expression [34]. In this study, we used the Western blot assay to examine changes in NOX4 expression to examine the effects of (+)-SYR (Figure 6a). The results indicated that α-MSH increased the expression of NOX4 while (+)-SYR attenuated them, suggesting that the antioxidant effects of (+)-SYR in melanocytes are attributable, at least in part, to the suppression of NOX4.

### 3.6. Effects of (+)-SYR on the Pigmentation in a Pigmented Human Skin Model, Melanoderm™

**Figure 7:**
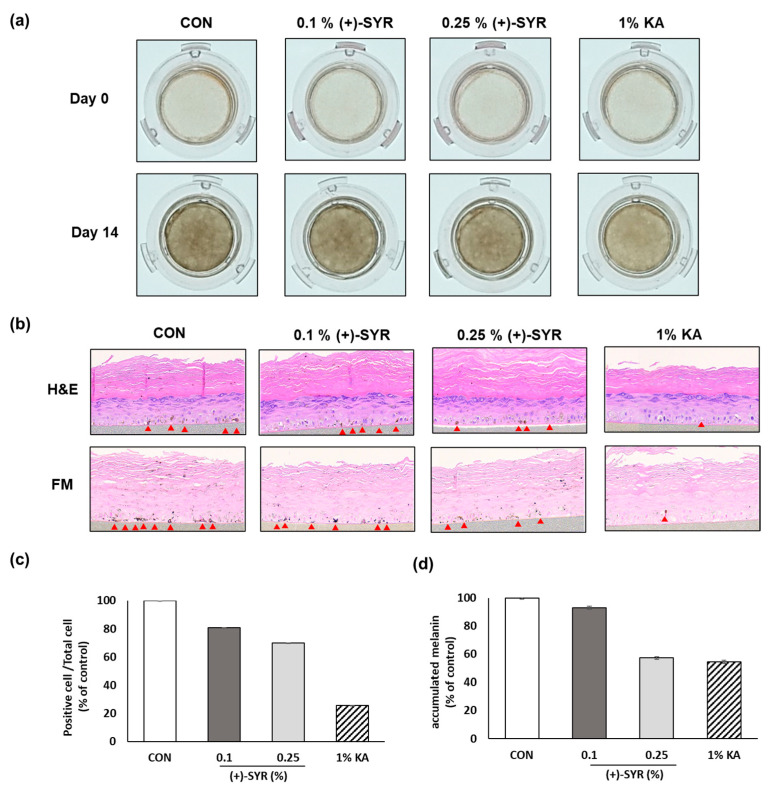
The effect of the (+)-SYR on the Melanoderm™, 3D human pigmented epidermis model. Melanoderm™ was treated with SYR (0.1%, 0.25%) and kojic acid (1%) every other day for 14 days. (**a**) Color change in the 3D human skin tissue model on Day 0 and Day 14; (**b**) tissues on Day 14 were stained with H&E and FM. Red arrowheads indicated the stained melanocyte. (**c**) Quantitative analysis of H&E-stained tissue and (**d**) quantitative analysis of FM-stained tissue for accumulated melanin by image J software (https://imagej.net/ij/, accessed on 1 September 2023).

The efficacy of (+)-SYR in brightening skin pigmentation was additionally assessed using Melanoderm™, an artificial model of the pigmented human epidermis, which provides an alternative to animal testing for research related to melanogenesis. After treatment with (+)-SYR, the skin tissue showed significant brightening compared to untreated skin (Figure 7a). On the last day of the experiment, the skin tissue was fixed and subjected to staining using H&E and Fontana–Masson (FM). The results showed the decreased melanin content and distribution (Figure 7b). Additionally, immunohistochemistry (IHC)-stained tissue revealed that (+)-SYR reduced the accumulation of melanin-containing organelles, known as melanosomes, and the formation of dendrites in melanocytes (Figure 7c,d). These findings suggest that (+)-SYR may effectively attenuate melanogenesis and skin pigmentation.

## 4. Discussion

The skin is frequently subjected to oxidative stress triggers from various origins, such as UV radiation and environmental pollution [35]. Increased levels of ROS in the skin can significantly contribute to skin issues, including hyperpigmentation, wrinkles, rough texture, and signs of aging [36,37]. Considering the pivotal role of ROS in melanin synthesis, scavenging ROS through antioxidant activity can effectively alleviate hyperpigmentation or decrease melanin production [38,39,40]. Supporting this, various kinds of antioxidants, including ascorbic acid (Vitamin C), ethyl ascorbyl ether, ascorbyl glucoside, and ascorbyl tetra-isopalmitate, are widely used as skin-brightening ingredients [10,41]. However, some antioxidants failed to show skin-brightening effects in vitro, possibly due to cell impermeability issues [42]. Our study confirmed that (+)-SYR suppressed melanin synthesis and the antioxidant activity of (+)-SYR inhibits both cytosolic ROS and mitochondrial ROS, demonstrating that (+)-SYR may serve as a new skin-brightening active antioxidant ingredient. 

ROS can arise from environmental factors like UV radiation and metabolic activities within mitochondria [25]. The prolonged exposure of the skin to both internal and external oxidative stressors leads to the overproduction of ROS, which may induce alterations in skin pigmentation [43]. Previous studies have shown that melanin production induced by ROS results from the increase in ROS levels in melanocytes exposed to UV radiation, leading to DNA damage. Moreover, ROS promotes the activity of p53, which regulates the activity of TYR and TRP-1 [44]. The primary origins of ROS in cells include mitochondria and NADPH oxidases (NOXs). ROS in the cytosol can originate from mitochondria or can be produced by NOX activity, whereas mitochondrial ROS stem from the oxidation of metabolic intermediates within the electron transport chain, with Complex I predominantly involved in this process [45]. 

NOXs are crucial for ROS production, influencing cell homeostasis and responses to stressors. They participate in redox-sensitive signaling pathways, but their overactivation can disrupt cellular balance, contributing to various diseases, aging, and cancer [46]. Zhao et al. demonstrated that inhibiting NOX activity with diphenyleneiodonium (DPI) in B16F10 melanoma cells results in reduced oxidative stress, which subsequently suppressed cell proliferation in skin carcinogenesis models. This suggests that NOX could serve as a viable target for antioxidant treatment [47]. Furthermore, NOX4 has been identified as a primary contributor to ROS production following α-MSH stimulation in B16F10 cells [48]. In our study, it was observed that (+)-(+)-SYR resulted in a more pronounced reduction in cytosolic ROS when compared with mitochondrial ROS, although previous studies showed (+)-SYR can have effects on the mitochondria [49,50]. Additionally, the decreased expression of NOX4 suggests that the antioxidant effect of (+)-SYR is more effective against cytosolic ROS than mitochondrial ROS. 

Melanoderm™, which has a 3D, structured epidermis similar to intact human skin, has been widely applied in numerous studies evaluating the brightening efficacy of cosmetics and pharmaceuticals. In this study, Melanoderm™ was utilized as a substitute for animal experiments to further confirm the brightening efficacy of (+)-SYR. Melanoderm™ has a stratum corneum (SC) layer, which is absent in the cell line system. Due to the presence of the SC layer, an incomplete skin absorption of the treated substances shall be considered when selecting the experimental concentrations. According to the manufacturer of Melanoderm™, the appropriate concentration of the positive control kojic acid was 2%, which is almost 200-fold higher than the effective concentrations observed in the cell line (~100 μM, 14 μg/mL). To consider this, the maximum concentration of (+)-SYR was set at 0.25%, which showed significant brightening effects. Given that kojic acid is used at concentrations from 1% to 2% in brightening cosmetics [51], it is expected that (+)-SYR can have a skin-brightening effect below 1%, although it must be proved through human studies in the future.

The elevation of melanin levels triggered by ROS is associated with the activation of the cAMP/PKA or MAPK signaling pathways. Specifically, studies have indicated that UV radiation exposure enhances MITF expression via the cAMP/PKA signaling pathway activation in melanocytes, ultimately resulting in increased melanin synthesis [52]. Moreover, it has been documented that ROS activate the MAPK signaling pathway, thereby enhancing melanin synthesis [53]. In this study, it was confirmed that (+)-SYR did not inhibit tyrosinase activity, but it did inhibit the expression of melanogenic enzymes, which may be attributable to the suppression of cytosolic ROS. However, it is necessary to examine the antioxidant capacity of (+)-SYR against other external stress factors like UV.

In a prior investigation, (+)-SYR was found to exhibit anti-aging properties by regulating antioxidant activity and autophagy [54]. Another study examined the anti-melanogenic and anti-aging effects of ginseng berry extract, demonstrating that (+)-SYR could decrease melanin production by activating antioxidant-FoxO3a signaling [8]. However, these studies have not touched on the anti-melanogenic mechanism with respect to melanogenesis. Furthermore, it was not known whether (+)-SYR reduces cytosolic or mitochondrial ROS. We demonstrated that the (+)-SYR reduces cytosolic ROS by inhibiting the activity of NOX4, which has contributed to anti-melanogenic efficacy, as determined by downregulated melanogenic enzymes and reduced dendrite extension.

In summary, our study confirmed the skin-brightening and antioxidant effects of (+)-SYR. (+)-SYR reduced melanogenesis by inhibiting the expression of TYR, TRP-1, and TRP-2, at both mRNA and protein levels. The antioxidant activity of (+)-SYR was confirmed through DPPH assay and DCFDA-ROS assay in B16F10 cells. Importantly, (+)-SYR decreased the α-MSH-induced expression of the NOX4 protein, suggesting that (+)-SYR’s antioxidant activity may involve the NADPH oxidase pathway. Finally, the skin-brightening effects of (+)-SYR were confirmed in a near in vivo artificial pigmented epidermis model, Melanderm™. Considering the increasing attention afforded to nature-friendly cosmetic ingredients, the anti-melanogenic effects and the antioxidant activity of (+)-SYR may provide important insights into the utilization of ginseng berry and its components for novel functional cosmetic ingredients. However, our findings on NOX4 are just observational using murine melanoma cells, and further molecular works to establish the causative relation between NOX4 suppression and skin brightening, and confirm it in human melanocytes, are necessary in the future.

## Data Availability

Data are available on request to the corresponding author.

## Supplementary Materials

The following supporting information can be downloaded at https://www.mdpi.com/article/10.3390/antiox13070876/s1, Figure S1: Western blot original image of NOX4; Figure S2: Western blot original image of TYR, TRP-1, TRP-2.
