# Supplementary material for: Syringaresinol Attenuates α-Melanocyte-Stimulating Hormone-Induced Reactive Oxygen Species Generation and Melanogenesis
Source: Antioxidants (Basel). 2024 Jul 21;13(7):876. doi: 10.3390/antiox13070876 (PMC11273534; doi:10.3390/antiox13070876)
Supplement: Supplementary file 1 [file antioxidants-13-00876-s001.zip › Figure S1.NOX4_Western blot Original image_n4.pdf]

NOX4

Predicted band size: 65 kDa

Observed band size: 65 kDa

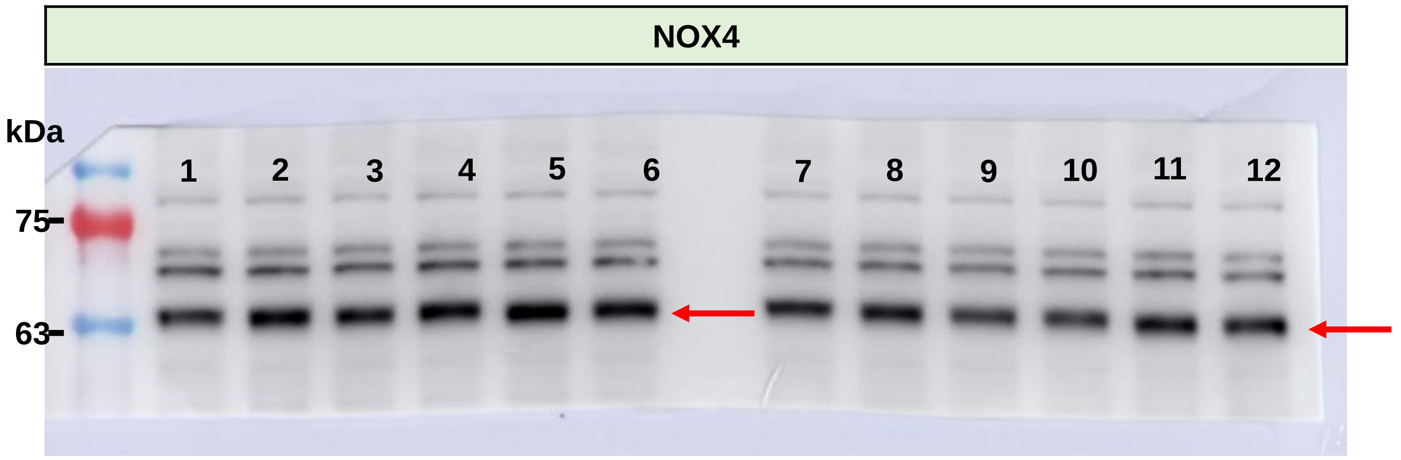

	n1						n2					
Time	5 min			10 min			5 min			10 min		
Lane #	1	2	3	4	5	6	7	8	9	10	11	12
α-MSH	-	+	+	-	+	+	-	+	+	-	+	+
(+)-SYR	-	-	+	-	-	+	-	-	+	-	-	+

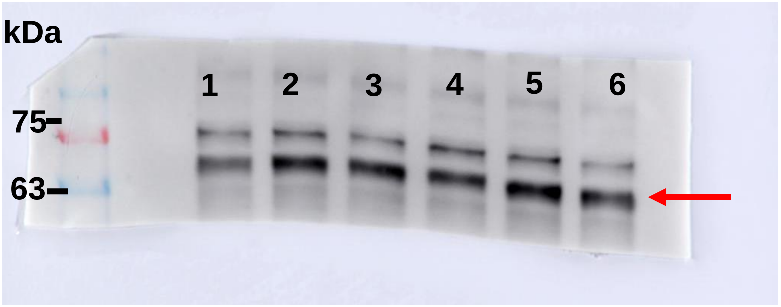

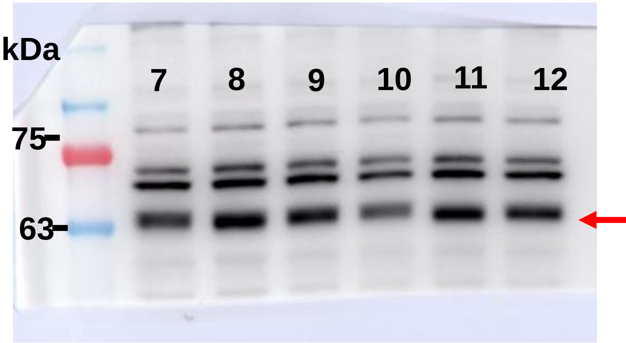

	n3						n4					
Time	5 min			10 min			5 min			10 min		
Lane #	1	2	3	4	5	6	7	8	9	10	11	12
α-MSH	-	+	+	-	+	+	-	+	+	-	+	+
(+)-SYR	-	-	+	-	-	+	-	-	+	-	-	+

GAPDH

Predicted band size: 36 kDa

Observed band size: 36 kDa

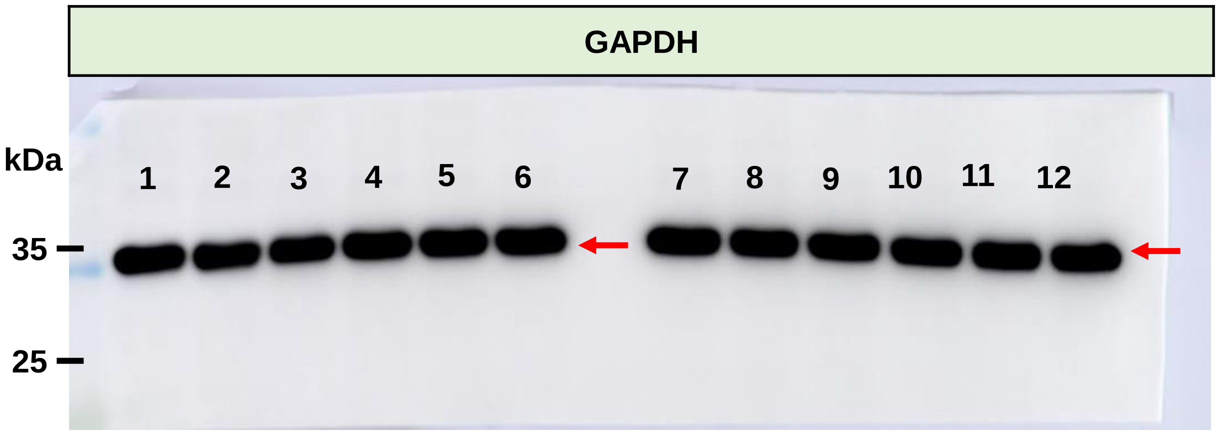

	n1						n2					
Time	5 min			10 min			5 min			10 min		
Lane #	1	2	3	4	5	6	7	8	9	10	11	12
α-MSH	-	+	+	-	+	+	-	+	+	-	+	+
(+)-SYR	-	-	+	-	-	+	-	-	+	-	-	+

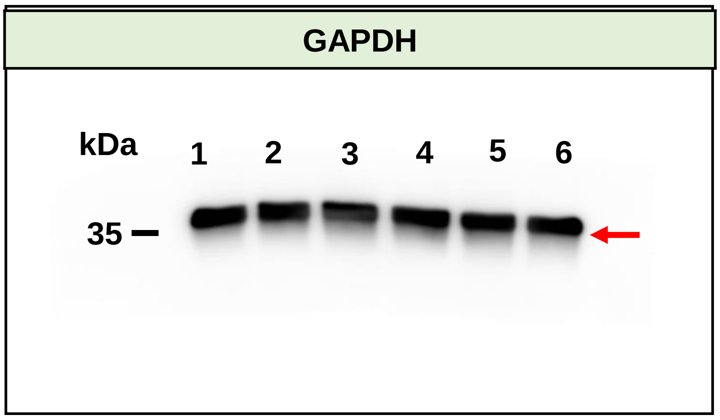

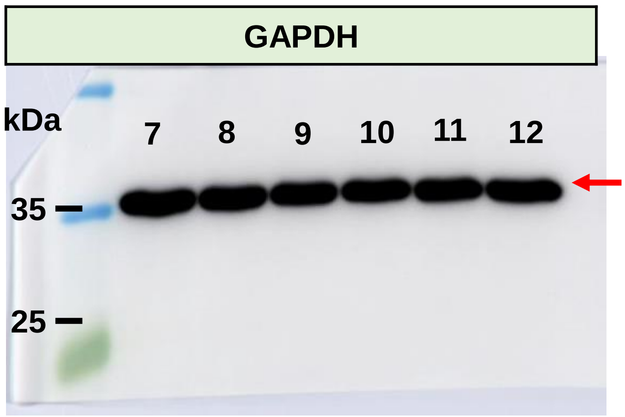

	n3						n4					
Time	5 min			10 min			5 min			10 min		
Lane #	1	2	3	4	5	6	7	8	9	10	11	12
α-MSH	-	+	+	-	+	+	-	+	+	-	+	+
(+)-SYR	-	-	+	-	-	+	-	-	+	-	-	+
