# Supplementary material for: Syringaresinol Attenuates α-Melanocyte-Stimulating Hormone-Induced Reactive Oxygen Species Generation and Melanogenesis
Source: Antioxidants (Basel). 2024 Jul 21;13(7):876. doi: 10.3390/antiox13070876 (PMC11273534; doi:10.3390/antiox13070876)
Supplement: Supplementary file 1 [file antioxidants-13-00876-s001.zip › Figure S2.TYR, TRP-1, TRP-2_Western blot Original image__n4.pdf]

TYR

Predicted band size: 70-80 kDa

Observed band size: 70 kDa

1)

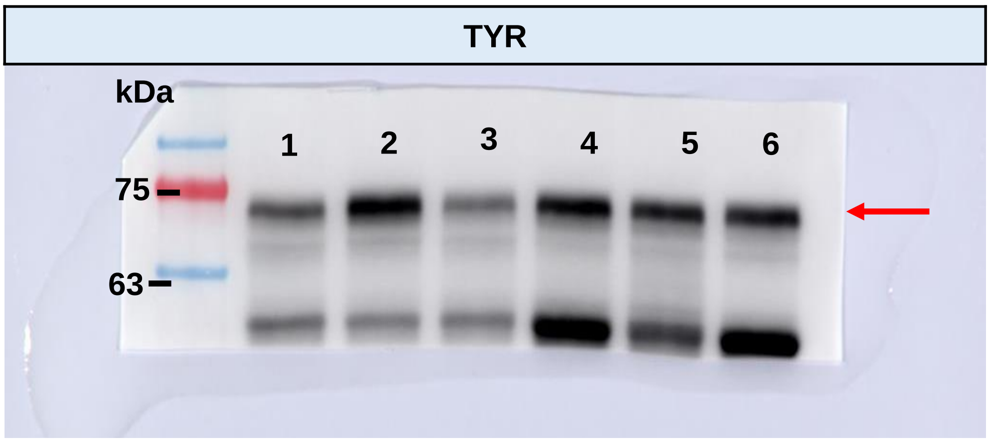

2)

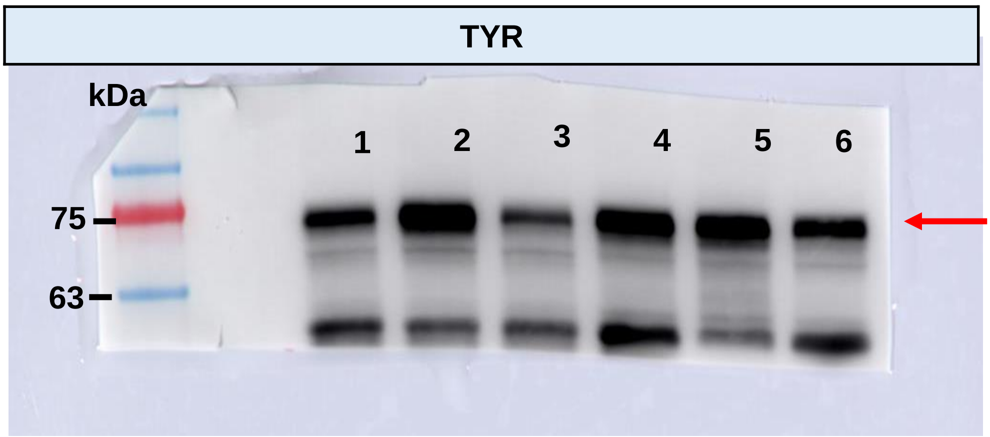

3)

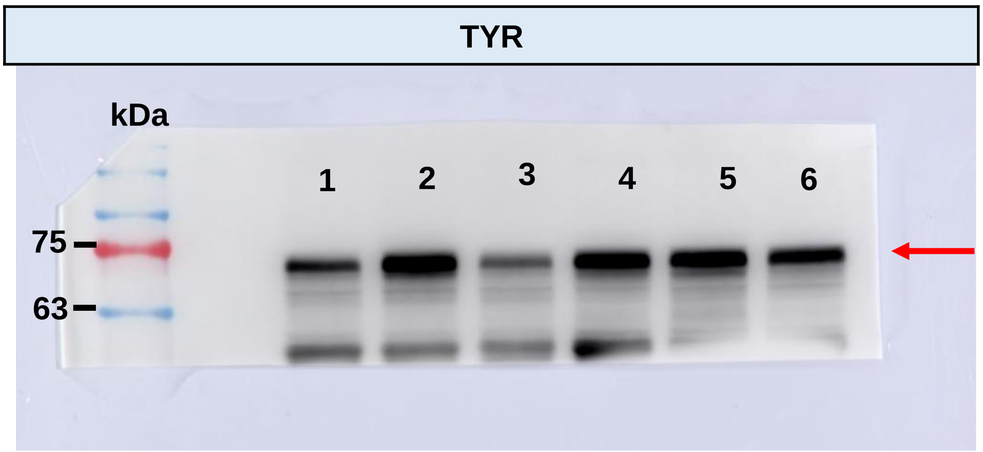

4)

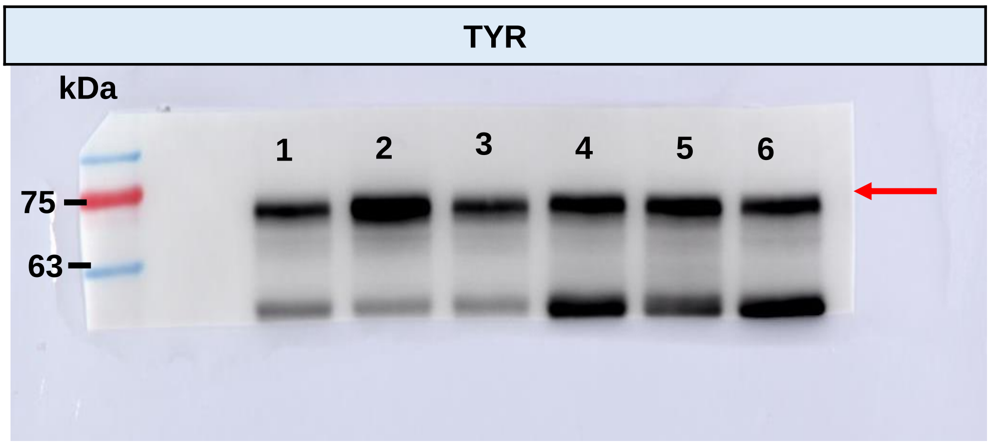

Time	24 h			48 h		
Lane #	1	2	3	4	5	6
α-MSH	-	+	+	-	+	+
(+)-SYR	-	-	+	-	-	+

TRP-1

Predicted band size: 70 kDa

Observed band size: 70 kDa

1)

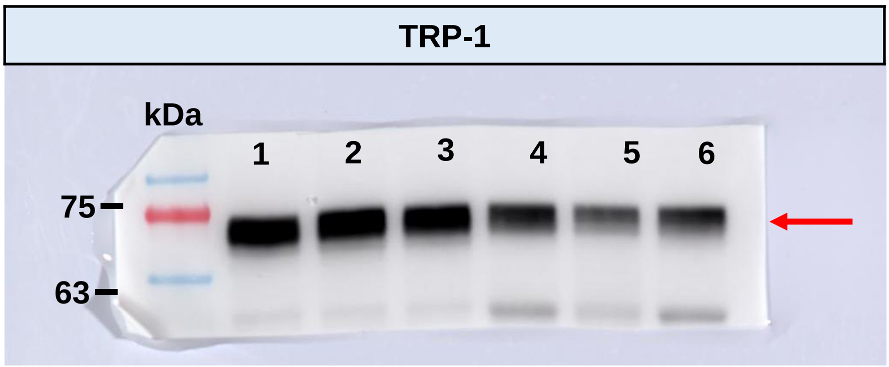

2)

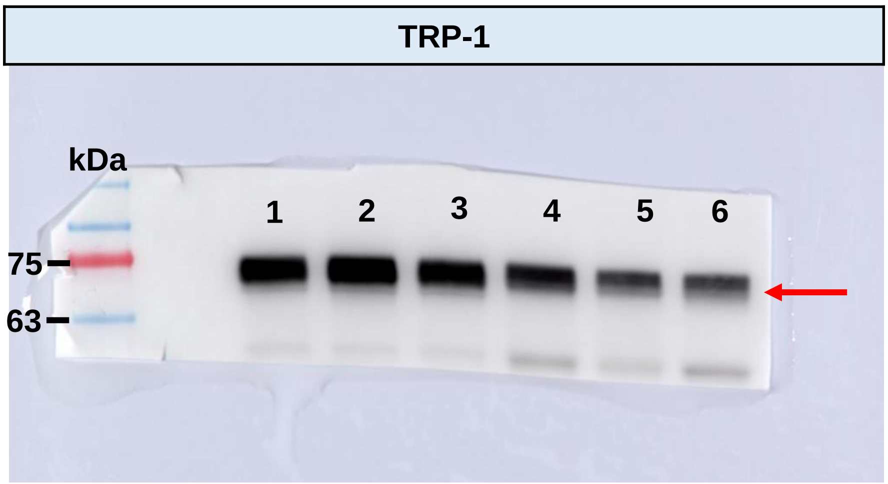

3)

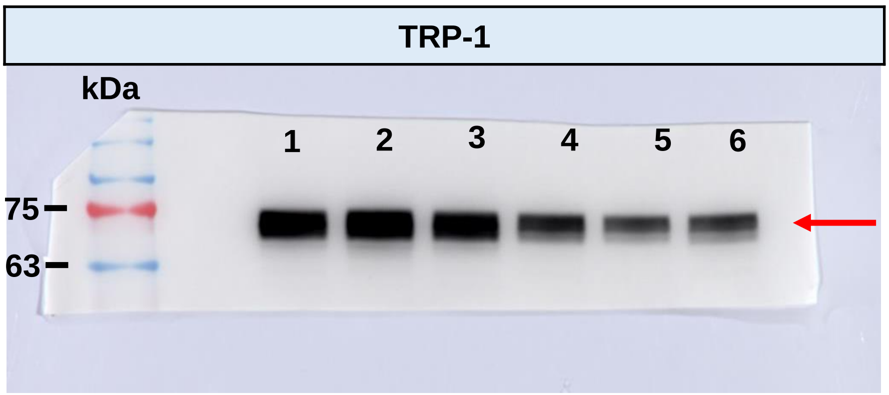

4)

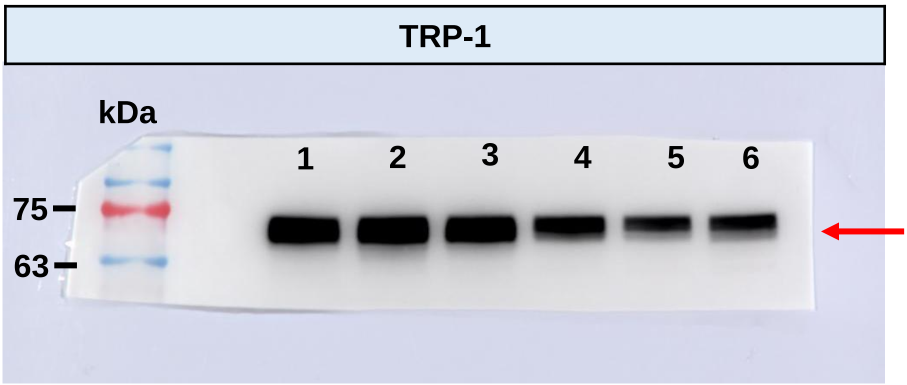

Time	24 h			48 h		
Lane #	1	2	3	4	5	6
α-MSH	-	+	+	-	+	+
(+)-SYR	-	-	+	-	-	+

TRP-2

Predicted band size: 70 kDa

Observed band size: 70 kDa

1)

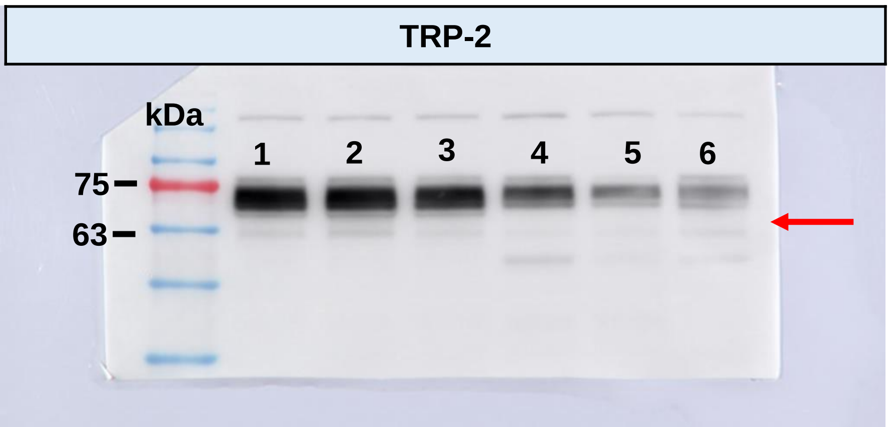

2)

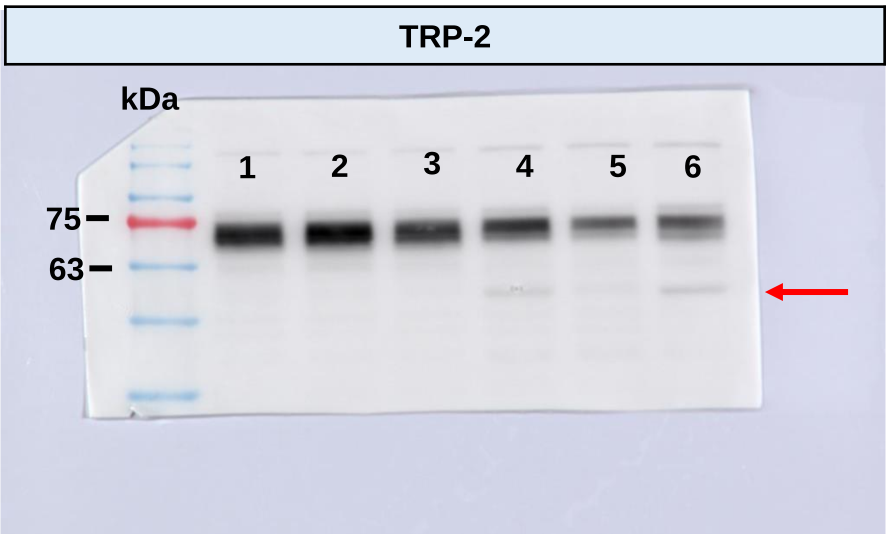

3)

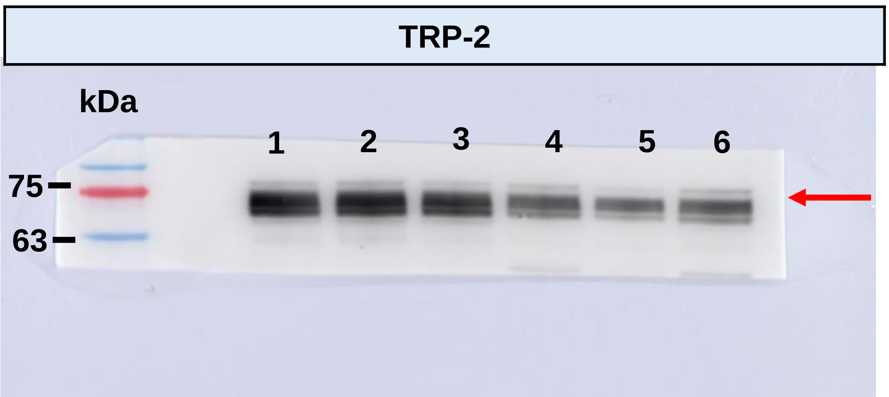

4)

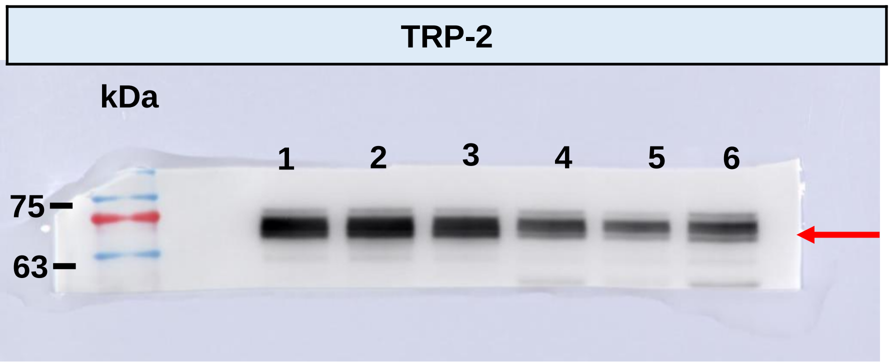

Time	24 h			48 h		
Lane #	1	2	3	4	5	6
α-MSH	-	+	+	-	+	+
(+)-SYR	-	-	+	-	-	+

b-actin

Predicted band size: 42 kDa

Observed band size: 42 kDa

1)

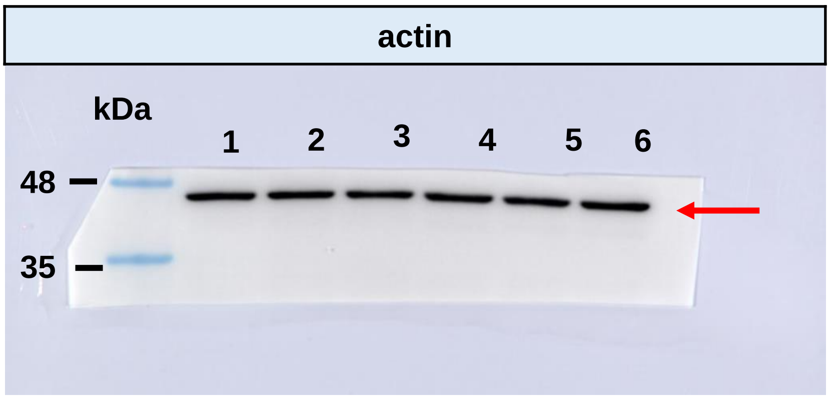

2)

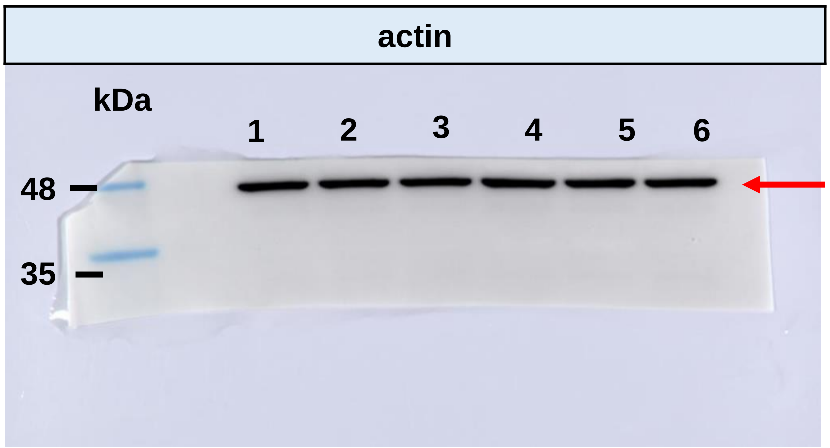

3)

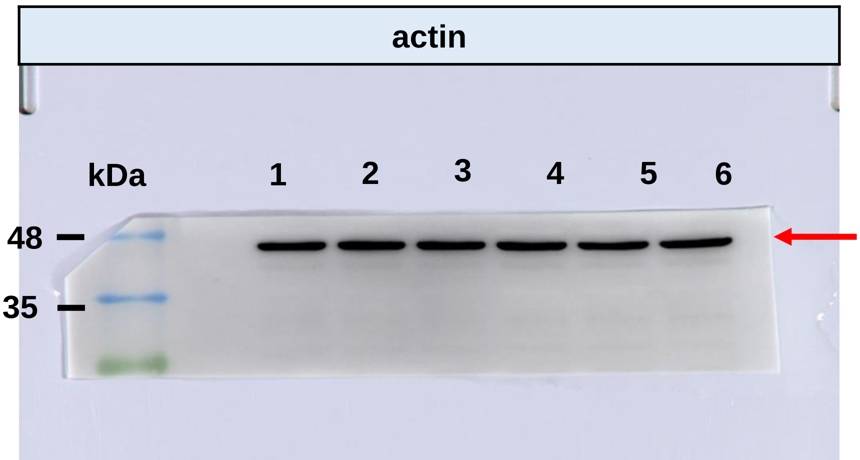

4)

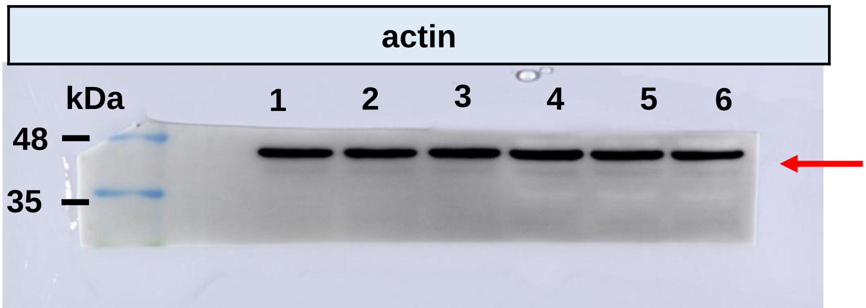

Time	24 h			48 h		
Lane #	1	2	3	4	5	6
α-MSH	-	+	+	-	+	+
(+)-SYR	-	-	+	-	-	+
